# Adaptation and implementation of a Nursing care protocol for children in the Amazon Region

**DOI:** 10.1590/0034-7167-2023-0245

**Published:** 2024-12-13

**Authors:** Aline Lara de Carvalho, Ezequiel Kleber Carpes Menezes, Deisi Cristine Forlin Benedet, Juliane Dias Aldrighi, Hérica de Lara Cardoso, Marcus Vinicius da Rocha Santos da Silva, Tatiane Herreira Trigueiro, Marilene Loewen Wall

**Affiliations:** I Secretaria Municipal de Saúde. Ji-Paraná, Rondônia, Brazil; II Secretaria de Estado da Saúde. Cacoal, Rondônia, Brazil; III Centro Universitário Internacional. Curitiba, Paraná, Brazil; IV Universidade Federal do Paraná. Curitiba, Paraná, Brazil; V Conselho Regional de Enfermagem. Curitiba, Paraná, Brazil

**Keywords:** Primary Health Care, Nursing Care, Child, Nursing, Protocol, Atención Primaria de Salud, Atención de Enfermería, Niño, Enfermería, Protocolo

## Abstract

**Objectives:**

to describe the process of implementing an adapted protocol for pediatric nursing care in a health unit located in a municipality in the Amazon Region.

**Methods:**

methodological research conducted in a basic health unit with four family health teams in the state of Rondônia, involving seven nursing professionals. Data collection occurred between October 2020 and April 2022, following the research phases: situational diagnosis, exploratory phase, protocol definition, implementation, and evaluation.

**Results:**

the outcome was the adaptation and implementation of a nursing care protocol for children.

**Final Considerations:**

the adaptation and implementation process can be an effective approach to improving care, strengthening nursing as a profession with a solid foundation in scientific and clinical evidence. This facilitates early problem identification and appropriate guidance, leading to better health outcomes for children.

## RESUMEN

**Objetivos:**

describir el proceso de implementación de un protocolo adaptado para el cuidado de enfermería infantil en una unidad de salud de un municipio en el interior de la Región Amazónica.

**Métodos:**

investigación metodológica, realizada en una unidad básica de salud con cuatro equipos de salud de la familia en el Estado de Rondônia, con siete profesionales de enfermería. La producción de datos se llevó a cabo entre octubre de 2020 y abril de 2022, de acuerdo con las fases de la investigación: diagnóstico situacional, exploratoria, definición del protocolo, implementación y evaluación.

**Resultados:**

el resultado fue la adaptación e implementación de un protocolo de cuidado de enfermería para niños.

**Consideraciones Finales:**

el proceso de adaptación e implementación puede ser un enfoque eficaz para mejorar el cuidado, fortaleciendo la enfermería como profesión con bases sólidas en evidencia científica y clínica. Esto permite la identificación temprana de problemas y la orientación adecuada, resultando en mejores resultados de salud para los niños.

## INTRODUCTION

Children belong to biologically and socially vulnerable groups, representing the majority of the population, with their uniqueness shaped by historical and social movements. As a result, they occupy an important place in the country’s public agenda^(1,2)^.

The health status of Brazilian children has significantly improved in recent decades due to better living conditions, the achievement of legal rights for children, and the promotion of public health policies. Additionally, important milestones have been reached, such as a decrease in infant mortality rates, expanded access to services, increased vaccination coverage, and a reduction in malnutrition^(2)^.

Primary Health Care (PHC) stands out in child care as a privileged setting for providing basic care through the adoption of clinical evidence to guide practice. This is supported by government policies, guidelines, protocols, and ministerial booklets, as well as by expanding the roles and actions of healthcare professionals. PHC is the main foundation that demonstrates its potential to improve the quality and efficiency of the healthcare system^(1,2)^.

In this context, the fundamental role of nurses in caring for the pediatric population across various healthcare sectors, especially in PHC, is highlighted by their contribution to targeted care practices. Moreover, through Nursing Consultations (NC) in PHC, a better quality of life for children can be promoted^(3)^, consequently contributing to the reduction of infant morbidity and mortality rates.

Furthermore, this research is justified by the absence of a protocol based on best practices for NC in child health in the Amazon Region, where it was conducted, as well as the lack of an implemented protocol. Thus, there is a need for a formal and scientific description of the nurse’s role in PHC in child care. It is important to emphasize the necessity for professionals to be able to identify risk factors, assess needs, and implement quality and resolute care, enabling them to provide differentiated care that meets demands, based on effective and welcoming assistance.

From this perspective, the research problem was defined as follows: how can a care protocol for nursing in child health be adapted and implemented in a primary healthcare unit?

## OBJECTIVES

To describe the process of implementing an adapted protocol for nursing care for children in a healthcare unit in a municipality in the interior of the Amazon Region.

## METHODS

### Ethical Aspects

This research is derived from a master’s thesis that implemented and adapted a protocol for nursing care for children in PHC. The study adhered to Resolution 466/12 of the National Health Council (CNS)^(4)^. The project was approved by the Research Ethics Committee of the Health Sciences Sector at the Federal University of Paraná (UFPR). Participants were fully informed about the research objectives and consented to participate by signing the Informed Consent Form.

### Study Design and Methodological Framework

Methodological Research (MR) was chosen for its potential to develop and validate new instruments, with the aim of testing the effect of a new protocol on the proportion of clarifying responses to solve a problem^(5-7)^. In this study, the implementation of a validated protocol was advocated to systematize nursing care for children in PHC^(5-7)^. The study’s writing was generally guided by the Standards for Reporting Qualitative Research (SRQR) instrument^(8)^. It is important to note that the research phases included situational diagnosis, an exploratory phase, protocol definition, implementation, and evaluation^(9,10)^.

### Study Setting

The research was conducted from October 2020 to April 2022 in a Basic Health Unit (BHU) located in the first district of the municipality of Ji-Paraná, Rondônia (RO), in the Amazon Region. The BHU, comprising four Family Health Strategy (FHS) teams, serves ten neighborhoods from Monday to Friday, from 7:30 AM to 5:30 PM. The team consists of five nursing technicians and four nurses. The service attends to about 300 people per day. Of this daily total, 28 are children aged 0 to 10 years, and there are approximately 1,550 children registered/affiliated with the BHU. The following sections describe the phases of research development that occurred to achieve the proposed objective.

### The First Phase: Situational Diagnosis

As a method for identifying and analyzing the local reality and its needs, the first author, a nurse at the BHU for six years, empirically identified the need for practical modifications to improve care through research. It was found that a significant proportion of child care visits resulted in unresolved issues, with weaknesses in care, deficiencies in decision-making, a lack of standardization in clinical practices, unified communication, and care coordination among professionals. This was primarily due to the absence of a nursing protocol to guide, support, and substantiate the care provided.

### The Second Phase: Exploratory

This phase involved searching for child health protocols validated by the Regional Nursing Councils (In Portuguese CORENs) across the country. The search began on November 18, 2020, with emails sent to all COREN offices inquiring about the existence of protocols and requesting authorization for their use in this research.

Many of the COREN offices acknowledged receiving the email but did not respond to the request, with only 11 out of the 27 COREN offices providing a response. Of these, only five had validated child health protocols, specifically: the Federal District, Paraíba, Paraná, Rio Grande do Sul, and Sergipe. The councils from Amazonas, Espírito Santo, Minas Gerais, and Pará informed that they were in the process of developing or validating their protocols and, therefore, did not grant authorization for use. Consequently, five protocols were analyzed to determine which best met the legal and ethical principles of the profession, the guidelines, norms, and regulations of evidence-based practice, as well as the national, state, and municipal health system standards, and the requirements of the institution that would implement it.

During this search for validated protocols, the following aspects were analyzed: clarity and comprehensibility of the texts and illustrations; relevance of the protocol’s content; year of publication/development; and relevance to the local reality. Additionally, the validation by the nursing team, specialists, and users was considered; whether the protocol was easy to read; whether it was based on evidence-based practice; whether it included a specific situation of care/assistance in its description; and specifications on what is done, who does it, and how it is done, guiding professionals in care decisions for the prevention, recovery, or rehabilitation of health^(11)^.

The existence of evaluation/diagnosis or care/treatment actions was noted, such as the use of educational interventions, treatments with physical means, and emotional, social, and pharmacological interventions, whether independent of nursing or shared with other professionals. Additionally, the suitability and grounding in documents provided by the Ministry of Health and the World Health Organization focused on PHC were assessed, as well as whether COREN authorized the implementation in Ji-Paraná.

### The Third Phase: Protocol Definition

After analyzing the existing protocols, the most up-to-date, comprehensive, suitable, and authorized protocol for implementation was selected in accordance with the Guidelines for the Development of the Nursing Process (NP) in PHC from the Federal Nursing Council (In Portuguese COFEN).

The aspects analyzed during data collection were fivefold: clarity and comprehensibility of the texts and illustrations; suitability; relevance of the protocol’s content; publication date; and relevance to the local reality. After thorough analysis, the “Nursing Protocol in Primary Health Care: Child Health Care”, adopted and implemented in the state of Paraná on December 9, 2020, was selected^(12)^.

### The Fourth Phase: Implementation

Thematic workshops were held with nursing professionals in both remote and in-person formats, using the Four Rs (RS) method, which includes the phases of Recognition, Revelation, Sharing, and Rethinking. This approach allowed the group to better understand the situation they were facing. A script was developed with the proposed themes and objectives to organize and guide each workshop. The workshops were titled: Weaknesses in Child Health Care; Systematizing Child Health Care; Experiencing the NP in Child Health; Agreement on the Child Health Protocol. These sessions facilitated reflection, discussion, sharing, and understanding among the nursing team throughout the process.

The choice of workshops represents an alternative in the pursuit of improvements, creating a complex time-space where participants are both actors and subjects who create forms of interaction that can transcend the critical application of theories and practices. In total, four workshops were held-two remotely and two in person-along with formal and informal meetings. The first workshop characterized the Recognition Phase, which preceded the group’s initiation. The goal was to present the research project to the participating professionals. The group presentation was conducted remotely.

The second workshop represented the Revelation Phase. The objective of this workshop was to develop pathways and consensus with participants to systematize child health care according to the defined protocol. In the third workshop, the Sharing Phase was addressed. This workshop was held in person at the BHU, lasted about 30 minutes, and aimed to share the experience of the protocol in daily practice. Participants were divided into groups to avoid disrupting the routine. In the fourth and final workshop, the Rethinking Phase was defined. This workshop also took place at the BHU and lasted about 30 minutes. It allowed for reflection on the content of the process through discussions and consensus with the nursing professionals after they had used the protocol during NC at the unit.

Out of a total of nine professionals, one team was without a nurse due to leave. The first author is a nurse at the BHU. Thus, seven nursing professionals participated: two nurses and five nursing technicians. The inclusion criteria were nursing professionals who provided child care at the BHU, the setting of this study. The exclusion criterion was nursing professionals on leave for various reasons during the research period.

Formal meetings were defined as those conducted via Microsoft Teams and in the BHU auditorium. Informal meetings took place during breaks between NCs at the BHU and through the WhatsApp tool. The implementation process occurred simultaneously with the adaptation process being discussed within the group by the participating professionals.

### The Fifth Phase: Evaluation

The results were analyzed and discussed simultaneously during the implementation workshops, recorded in a field diary through participant observation and the participants’ statements, which were gathered through the sharing of experiences, insights, and suggestions during the implementation process. Participants’ perceptions of the protocol and the adaptation process were evaluated using Google Forms. Upon analyzing the results, a favorable opinion toward the implementation of the Nursing Protocol was observed. Finally, the final version was submitted for evaluation by the Department of Primary Care of the Municipal Health Secretariat and was approved for implementation in the BHUs of the municipality.

### Analysis

The methodological approach to analysis followed the framework proposed by Creswell^(13)^. Subsequently, the data discussed in the workshops were fully transcribed according to the development of each workshop, identified, and analyzed according to the themes and objectives proposed, using Microsoft Word. Each participant was identified by letters and numbers to ensure anonymity.

The synthesis aimed to compare the concepts extracted from the participants’ statements, using different colors for each participant based on the identified concepts. The data were compared, contextualized, and organized according to the participants’ accounts of ideas, concepts, events, and facts. Considering the information collected during the synthesis, four categories emerged. For each category, derived from the in-depth analysis, the discussion was supported by scientific literature.

## RESULTS

To adapt the protocol, it was necessary to explore the topic with the group to provide both theoretical and practical support among healthcare professionals. Among the participants, the majority were female, with six women and one man. The duration of service in the position and at the BHU varied: one professional had more than 30 years of experience, four had more than 20 years, and two had between 5 and 15 years of experience in the healthcare field, with an average of 24.2 years working in the FHS. The range of experience varied from 24 months to 35 years.

After comparing the identified information, four categories were established: Experiences with the protocol in daily practice; Weaknesses and limitations in the implementation process; Facilitators in the implementation process; and Suggestions for adapting the protocol to the reality of Rondônia (RO).

From the perspective of the research participants, the category titled “Experiences with the Protocol in Daily Practice” suggests an improvement in the quality of health services offered to children in PHC, enabling better team integration, which results in welcoming the mother/user and her child, the effective conduct of NC, humanization of care, addressing unmet demand, and ultimately, integrating theory with practice.

The category “Weaknesses and Limitations in the Implementation Process” refers to the everyday impact of demands during NCs, as well as the limitations of access within the SUS framework. It emphasizes the context, established strategies, certain determinants and contributing factors, inadequate staffing levels in the nursing team, insufficient time for study, and concerns about expanding the protocol to all BHU.

The category “Facilitators in the Implementation Process” describes the protocol as a facilitating tool for implementing the systematization of nursing care (SNC), promoting improvements in work processes in the pursuit of care excellence. Additionally, the use of audiovisual resources to support the process and the adaptation of a protocol already validated by COREN/PR effectively facilitated implementation.

Finally, the category “Suggestions for Adapting the Protocol to the Municipality’s Reality” discusses the sharing of knowledge and analysis of nursing professionals’ perceptions to improve the final product. As a result, the adaptation process of the Nursing Protocol in PHC: Module 4 - COREN/PR was tailored to the reality of the health unit in Ji-Paraná.

### Protocol Adaptation Process

Through the adaptation process, a new product was developed. This was restructured to fit the local reality with contributions from professionals during the meetings. The original protocol contained eight chapters, all of which underwent adaptations, with subchapters added along with 11 annexes, as shown in Chart 1.

**Chart 1:** Comparison between the original protocol and the adapted protocol, Ji-Paraná, Rondônia, Brazil, 2023

Original Protocol	Adapted Protocol
1. Nurse Consultation for Monitoring the Growth and Development of a Healthy Child. 2. Nurse Consultation for Monitoring the Growth and Development of a Child with Special Care Needs. 3. Evaluation of a Child with Respiratory Problems 3.1 Ear Pain 4. Evaluation of a Child with Nutrition or Feeding Problems 5. Evaluation of a Child with Diarrhea and/or Dehydration 6. Evaluation of a Child with Anemia 7. Evaluation of a Child with Eye Problems 8. Evaluation of a Child with Dermatological Problems	1. Nurse Consultation for Monitoring the Growth and Development of a Healthy Child 2. Nurse Consultation for Monitoring the Growth and Development of a Child with Special Care Needs 3. Evaluation of a Child with Respiratory Problems 3.1. Ear Pain 4. Evaluation of a Child with Nutrition or Feeding Problems 5. Evaluation of a Child with Diarrhea and/or Dehydration 5.1. Procedures to be Adopted in Cases of Dysentery and/or Other Pathologies Associated with Diarrhea 5.2. Epidemiological Surveillance of Acute Diarrheal Diseases (ADD) 6. Evaluation of a Child with Anemia 6.1. Laboratory Diagnosis 6.2. Treatment 7. Evaluation of a Child with Eye Problems 8. Evaluation of a Child with Dermatological Problems

Chart 2 provides a concise description of the adaptations and justifications for each item according to each chapter.

**Chart 2:** Summary of the adapted protocol, Ji-Paraná, Rondônia, Brazil. 2023

Chapter 1. Nurse Consultation for Monitoring the Growth and Development of a Healthy Child	
**Included in the Adaptation**	**Justification**
The specific responsibilities of nursing professionals and community health agents regarding child care.	Clarify the responsibilities of each professional
Inserted tables: Representation of the child’s reflexes; representation of the Barlow and Ortolani tests; Low-Risk Child Consultations; Updated risk stratification; expanded request for exams.	Enhance and facilitate access to information, as well as clearly specify the exams that professionals can request.
Added to Table 9: Adverse Events Post-Vaccination; Congenital Nasolacrimal Duct Obstruction; Umbilical Hernia; Inguinal Hernia; Cryptorchidism; Hydrocele; Phimosis; Umbilical Stump; Umbilical Granuloma; Reflux; Management of Umbilical Granuloma in the Absence of Silver Nitrate Stick.	Simplify the procedures for professionals
**Chapter 2. Nurse Consultation for Monitoring the Growth and Development of a Child with Special Care Needs**	
Inserted Table: Classification of the Newborn. Flowchart: Nursing Consultation for a Child with Dehydration	Facilitate access to information
**Chapter 3. Evaluation of a Child with Respiratory Problems**	
Inserted Tables: Evaluation of Difficulty Breathing; Management of Cough or Difficulty Breathing.	Facilitate access to information
**Chapter 3.1 Ear Pain**	
Inserted Tables: Evaluation and Classification of Children with Ear Pain; Findings on Otoscopy.	Facilitate access to information
**Chapter 4. Evaluation of a Child with Nutrition or Feeding Problems**	
Inserted Tables: Evaluation of Malnutrition According to IMCI Brazil^(14)^; Management of Child Growth; Prophylaxis - Administration of Vitamin A in Children; Anthropometric Gain - 1st to 2nd Year.	Facilitate access to information
**Chapter 5. Evaluation of a Child with Diarrhea and/or Dehydration**	
Inserted Tables: Diarrheal Characteristics in Children Under 2 Years Based on the Text Described by the Authors; Management During Treatment in Children.	Facilitate access to information
**Chapter 6. Evaluation of a Child with Anemia**	
The text at the beginning of the chapter was summarized. Flowchart 7 was adapted to the local context.	Facilitate access to information
**Chapter 7. Evaluation of a Child with Eye Problems**	
Inserted Table - Eye Problems	Facilitate access to information
**Chapter 8. Evaluation of a Child with Dermatological Problems**	
Oral Thrush with Images; Erythematous Intertrigo; Fungal Infection; Tinea Corporis; Insect Bites (Strophulus); Chickenpox; Hand-Foot-Mouth Syndrome; Non-Bullous Impetigo; Furunculoid Myiasis; Cutaneous Larva Migrans	Facilitate access to information
**Protocol Annexes**	
Growth Curve by Age from Birth to 5 Years; Developmental Milestones in the First 5 Years; Intercurrences and Management in Pediatric Care for Special Situations; Routine Vaccination Schedule from the Ministry of Health; Neonatal Jaundice; Promoting Oral Health.	Facilitate access to information

*IMCI - Integrated Management of Childhood Illness.*

## DISCUSSION

Protocols aim to contribute to appropriate and effective care, ensuring efficacy for the clinical condition and providing more benefits than harm. Additionally, they aim to assist in decision-making, constituting a unique care situation with detailed operational specifications^(15)^. In this context, upon identifying inadequacies in standardized care decision-making during the diagnostic phase, we reached a consensus that professionals should follow evidence-based protocols when caring for children.

Protocols are increasingly becoming widely used tools in promoting evidence-based nursing, allowing nurses to implement scientific knowledge in their practice, thereby improving the quality and safety of care^(16)^. Various protocol models exist, focusing on “soft-hard” technology and organized according to their practical content. The most significant difference among them is their applicability^(15)^. During the investigation phase, we found that only eleven protocols were validated and had access to the provided care. After evaluating the elements related to eligibility, the available documents were reviewed, and approval for implementation was granted. In the third phase, after data collection and thorough analysis, the protocol to be adapted was defined.

The implementation phase involved conducting thematic workshops using the Four Rs method. As a result, the protocol was successfully adapted. The literature indicates that the use of protocols benefits nurses’ performance by guiding their therapeutic actions. This observation is based on the ability of protocols to summarize the therapeutic process, reduce the time spent on documenting professional conduct, and complement the systematization of information available for continuous nursing care^(17)^.

Thus, care and assistance protocols, standard operating procedures, and other tools are essential for guiding nurses in patient care. They should be applied in various healthcare settings to ensure quality care^(18)^. During the workshops, the nursing team was made aware of the importance of the topic, received explanations on how the protocol would be applied, and engaged in periodic discussions with the researcher about doubts, negative and positive points, and suggestions after beginning to use the protocol during consultations.

A systematic review observed that few scientific studies identify and assess nurses’ skills in child healthcare. The research included 18 studies from six countries and described nurses’ clinical skills in childhood. However, the results do not provide a solid foundation for evaluating the scope of their practices. Health education and counseling, health and developmental assessments of children and adolescents, immunizations, and child health screenings were the most commonly identified, described, and independently performed nursing competencies^(19)^.

Regarding the interaction among professionals, during the implementation process of the protocol, the research participants, enthusiastic about the initiative, provided suggestions and applied the protocol during NCs. When encountering a real situation in child health during the NCs that was not described in the protocol, the professional would document it, and then all questions would be discussed as a team to adapt the protocol through a process of joint decision-making. This fostered a relationship between team members and users, promoting dialogical, empathetic, and ethical communication that encouraged autonomy, cooperation, and shared responsibility among the residents. This contribution to practice is supported by a study conducted in Recife-PE^(20)^.

During this period, informal contacts with the entire health team revealed the outcomes of the consultations guided by the protocol. The application of the protocol in the NCs increased the demand for nursing services in the unit. Thus, participatory planning evidently contributed to changes in the Primary Health Care Unit where it was implemented. Additionally, it has the potential to drive institutional changes throughout the municipal health sector regarding child health.

This finding aligns with the literature, which indicates that the pursuit of quality in nursing actions and collective dialogic construction drive the development of local protocols. Recognizing protocols as decision-support tools that provide technical and ethical guidance contributes to the advancement of knowledge and the development of effective nursing practices^(21)^.

From this perspective, the evaluation phase, which involves a collective and dialogic construction process, along with the adaptation of protocols to the local context, served as an important exercise in ongoing education and deepening the technical-scientific knowledge of nurses, with active participation from various stakeholders in the learning process^(21)^.

Thus, this study demonstrates how professionals recognize that establishing care protocols aids in organizing and structuring work processes, thereby enhancing care quality.

In this context, it is essential to reflect on and reassess the range of products, such as protocol development, that are vital for nursing care. Moreover, the rich implementation process, which integrates theoretical knowledge with practical experience, should be emphasized as a trend to be developed within the work environment. This process requires the competence of nurses working in PHC to effectively guide their practice.

### Study limitations

Several important limitations of the study should be noted. These include the SARS-CoV-2 pandemic, which introduced barriers and social distancing measures to prevent the spread of COVID-19. During the most critical phase of the pandemic, the unit continued operating, and the workshops, initially planned to be conducted in person, were reorganized into a remote format. Another limiting factor was the lack of available time for nursing professionals to meet, in addition to their increased workload.

### Contributions to the Field

The adaptation of the protocol to the local context brought benefits to health and nursing care, such as increased safety for nurses and a higher number of NC for child health. The nursing protocol fosters professional knowledge, skills, and attitudes, enabling the adoption of evidence-based practices and providing safe and qualified care for children in PHC in the Amazon Region, with positive impacts on their growth and development.

## FINAL CONSIDERATIONS

The adaptation and implementation of the nursing care protocol for child health in PHC were successful, bringing significant benefits. This process allowed nurses, in collaboration with the team, to share scientific knowledge, facilitating safe, high-quality, evidence-based care. Improvements were noted at each stage of the protocol’s application in nursing care, resulting in better health care for children, enhanced team integration, and improved service for mothers and children. The methodological research adjusted the protocol to the local context and facilitated its implementation by gathering information during thematic workshops. Initially implemented in one health unit, it demonstrated high potential for municipal-wide institutionalization due to its practical applicability.

The activities related to child monitoring, along with the learning and experience gained after the workshops, were confirmed by the positive feedback from participants. We have shown that the adaptation and implementation process is an effective approach for improving care, strengthening nursing as a profession with solid evidence-based and clinical foundations. This allows for early problem identification and appropriate guidance, resulting in better health outcomes for children. It is anticipated that this study will contribute to the creation of new data and scientific evidence in the field.
