# Supplementary material for: Adaptation and implementation of a Nursing care protocol for children in the Amazon Region
Source: Rev Bras Enferm. 2024 Dec 13;77(5):e20230245. doi: 10.1590/0034-7167-2023-0245 (PMC11654223; doi:10.1590/0034-7167-2023-0245)
Supplement: Translation

# Translation: Adequação e implantação de um protocolo de cuidados de Enfermagem para crianças na Região Amazônica

**DOI:** 10.1590/0034-7167-2023-0245pt

**Authors:** Aline Lara de Carvalho, Ezequiel Kleber Carpes Menezes, Deisi Cristine Forlin Benedet, Juliane Dias Aldrighi, Hérica de Lara Cardoso, Marcus Vinicius da Rocha Santos da Silva, Tatiane Herreira Trigueiro, Marilene Loewen Wall

**Affiliations:** I Secretaria Municipal de Saúde. Ji-Paraná, Rondônia, Brasil; II Secretaria de Estado da Saúde. Cacoal, Rondônia, Brasil; III Centro Universitário Internacional. Curitiba, Paraná, Brasil; IV Universidade Federal do Paraná. Curitiba, Paraná, Brasil; V Conselho Regional de Enfermagem. Curitiba, Paraná, Brasil

**Keywords:** Atenção Primária à, Saúde, Cuidados de Enfermagem, Criança, Enfermagem, Protocolo

## Abstract

**Objetivos:**

descrever o processo de implantação de um protocolo adaptado para o cuidado de enfermagem à criança em uma unidade de saúde de um município do interior da Região Amazônica.

**Métodos:**

pesquisa metodológica, realizada em uma unidade básica de saúde com quatro equipes de saúde da família do Estado de Rondônia, com sete profissionais de enfermagem. A produção dos dados ocorreu entre outubro de 2020 e abril de 2022, conforme fases da pesquisa: diagnóstico situacional, exploratória, definição do protocolo, implantação e avaliação.

**Resultados:**

o resultado foi a adaptação e implantação de um protocolo de cuidado de enfermagem à criança.

**Considerações Finais:**

o processo de adaptação e implantação pode ser uma abordagem eficaz para melhorar o cuidado, fortalecendo a enfermagem como profissão com bases sólidas em evidências científicas e clínicas. Isso permite a identificação precoce de problemas e a orientação adequada, resultando em melhores desfechos de saúde para a criança.

## INTRODUÇÃO

As crianças pertencem a grupos de vulnerabilidade biológica e social, expressando-se como maioria ao nível populacional, com sua singularidade permeada por movimentos históricos e sociais. Consequentemente, ocupam um lugar importante na agenda pública do país^(1,2)^.

O estado de saúde das crianças brasileiras melhorou significativamente nas últimas décadas devido a melhorias nas condições de vida da população, conquistas dos direitos legais das crianças e promoção de políticas públicas de saúde. Além disso, marcos importantes foram alcançados, tais como: queda nos índices de mortalidade infantil, expansão do acesso aos serviços, maior cobertura vacinal e redução da desnutrição^(2)^.

No cuidado à criança, destaca-se a Atenção Primária à Saúde (APS), local privilegiado para a efetivação dos cuidados básicos, por meio da adoção de evidências clínicas para nortear a prática, amparada em políticas governamentais, diretrizes, protocolos e cadernos ministeriais, bem como na ampliação de funções e ações dos profissionais de saúde, como principal alicerce que demonstra seu potencial para melhorar a qualidade e eficiência do sistema de saúde^(1,2)^.

Nesse sentido, sobressai o papel fundamental do enfermeiro no atendimento à população infantil nos diversos setores de saúde, em especial na APS, por contribuir com práticas direcionadas de cuidado. Além disso, com a Consulta de Enfermagem (CE) na APS, pode-se promover uma melhor qualidade de vida às crianças^(3)^, e, consequentemente, favorecer ativamente a redução das taxas de morbimortalidade infantil.

Ademais, a presente pesquisa justifica-se, pois, na Região Amazônica em que foi desenvolvida, não há protocolo fundamentado nas boas práticas para a CE à saúde da criança, tampouco implantado. Dessa forma, faz-se necessária a descrição formal e científica da atuação do enfermeiro na APS no cuidado à criança, sendo importante ressaltar a necessidade de o profissional estar apto a identificar os fatores de risco, avaliar necessidades para implementar um cuidado de qualidade e resolutivo, possibilitando, dessa forma, prestar um cuidado diferenciado, que supra as demandas, baseado em assistência eficaz e acolhedora.

Sob essa perspectiva, definiu-se a questão-problema da pesquisa: como adaptar e implantar um protocolo assistencial para o cuidado de enfermagem à criança em unidade básica de saúde?

## OBJETIVOS

Descrever o processo de implantação de um protocolo adaptado para a assistência de enfermagem à criança em uma unidade de saúde de um município do interior da Região Amazônica.

## MÉTODO

### Aspectos Éticos

A presente pesquisa é oriunda de uma dissertação de mestrado que implantou e adequou um protocolo de cuidado de enfermagem à criança na APS. O estudo seguiu a Resolução 466/12 do Conselho Nacional de Saúde (CNS)^(4)^. O projeto foi aprovado pelo Comitê de Ética em Pesquisa do Setor de Ciências da Saúde da Universidade Federal do Paraná (UFPR). Os participantes foram informados e esclarecidos quanto aos objetivos da pesquisa e consentiram em participar mediante assinatura do Termo de Consentimento Livre e Esclarecido.

### Desenho do Estudo e Referencial Metodológico

Optou-se pela Pesquisa Metodológica (PM) em razão de possibilitar o desenvolvimento e a validação de novos instrumentos, com o intuito de testar o efeito de um novo protocolo sobre as proporções de respostas esclarecedoras na solução de um problema^(5-7)^. Nesta pesquisa, foi defendida a implantação de um protocolo validado para sistematizar o cuidado de enfermagem à criança na APS^(5-7)^.

A redação do estudo foi orientada de forma genérica pelo instrumento *Standards for Reporting Qualitative Research* (SRQR)^(8)^. Ressalta-se que as fases da pesquisa compreenderam: diagnóstico situacional, exploratória, definição do protocolo, implantação e avaliação^(9,10)^.

### Cenário do Estudo

A pesquisa foi realizada no período de outubro de 2020 a abril de 2022, em uma Unidade Básica de Saúde (UBS), no primeiro distrito do município de Ji-Paraná, Rondônia (RO), Região Amazônica. A UBS, composta por quatro equipes da Estratégia de Saúde da Família (ESF), atende dez bairros, de segunda a sexta-feira, das 7h30 às 17h30. A equipe conta com cinco técnicos de enfermagem e quatro enfermeiras. O serviço atende cerca de 300 pessoas por dia. Desse total diário, 28 são crianças de 0 a 10 anos, e há cerca de 1550 crianças cadastradas/vinculadas na UBS. A seguir, serão descritas as fases de desenvolvimento da pesquisa para alcançar o objetivo proposto.

### A Primeira Fase: Diagnóstico Situacional

Como método de identificação e análise da realidade local e suas necessidades, a primeira autora, enfermeira na UBS há 6 anos, identificou, empiricamente, a necessidade de modificações práticas para a melhoria dos cuidados por meio da pesquisa. Constatou uma proporção significativa de atendimentos às crianças que retornavam com demandas não resolvidas, com fragilidade na assistência, precariedade na tomada de decisão do cuidado, falta de padronização de condutas clínicas, comunicação unificada e coordenação do cuidado prestado pelos profissionais, devido à ausência de um protocolo de enfermagem para guiar, respaldar e fundamentar o cuidado.

### A Segunda Fase: Exploratória

Essa fase consistiu na busca por protocolos na área de saúde da criança, validados pelos Conselhos Regionais de Enfermagem (CORENs) de todo o país. A busca iniciou-se em 18 de novembro de 2020, com o envio de e-mails a todos os CORENs, inquirindo-os sobre a existência de protocolos e solicitando autorização para sua utilização nesta pesquisa. Muitos dos CORENs reconheceram o recebimento do e-mail, mas não responderam à solicitação, sendo que apenas 11 dos 27 CORENs o fizeram. Destes, somente cinco possuíam protocolo de saúde infantil validado, sendo eles: Distrito Federal, Paraíba, Paraná, Rio Grande do Sul e Sergipe. Os conselhos do Amazonas, Espírito Santo, Minas Gerais e Pará informaram estar em fase de desenvolvimento ou validação e, portanto, não deram autorização para uso. Assim, cinco protocolos foram analisados, buscando-se encontrar aquele que atendesse aos princípios legais e éticos da profissão, às diretrizes, normas e regulamentos da prática baseada em evidências e às normas e regulamentos do sistema de saúde nacional, estadual e municipal, bem como da instituição que o utilizaria.

Nesta etapa de busca por protocolos validados, foram analisados os seguintes aspectos: clareza e inteligibilidade dos textos e ilustrações; relevância do conteúdo do protocolo; ano de publicação/elaboração; e relevância para a realidade local. Além disso, também foram observados a validação pela equipe de enfermagem, especialistas e usuários; se era de fácil leitura; se estava amparado em práticas baseadas em evidências; se continha em sua descrição uma situação específica de assistência/cuidado; especificações sobre o que se faz, quem faz e como se faz, conduzindo os profissionais nas decisões de assistência para a prevenção, recuperação ou reabilitação da saúde^(11)^.

Foi observada a existência das ações de avaliação/diagnóstico ou de cuidado/tratamento, como o uso de intervenções educacionais, tratamentos com meios físicos, intervenções emocionais, sociais e farmacológicas, independentes de enfermagem ou compartilhadas com outros profissionais, bem como se possuíam adequabilidade e embasamento nos documentos disponíveis pelo Ministério da Saúde e Organização Mundial da Saúde, voltados para a APS e se o COREN autorizava a implantação em Ji-Paraná.

### A Terceira Fase: Definição do Protocolo

Após a análise dos protocolos existentes, optou-se por selecionar o produto mais atualizado, completo, adequado e autorizado para implantação, conforme as Diretrizes de Desenvolvimento do Processo de Enfermagem (PE) na APS do Conselho Federal de Enfermagem (COFEN).

Os aspectos analisados durante a coleta de dados foram cinco: clareza e inteligibilidade dos textos e ilustrações; aptidão; relevância do conteúdo do protocolo; data de publicação; e relevância para a realidade local. Após uma análise aprofundada, foi definido o “Protocolo de Enfermagem na Atenção Primária à Saúde: Atenção à Saúde da Criança”, adotado e implantado no estado do Paraná em 9 de dezembro de 2020^(12)^.

### A Quarta Fase: Implantação

Foram realizadas oficinas temáticas com os profissionais de enfermagem nos formatos remoto e presencial, utilizando o método Quatro Erres (RS), que compreende as Fases de Reconhecimento, Revelação, Repartir e Repensar. Dessa forma, foi possível compreender a situação enfrentada pelo grupo. Foi elaborado um roteiro com os temas e objetivos propostos para fins de organização e desenvolvimento de cada oficina. Foram intituladas: Fragilidades na Atenção à Saúde da Criança; Sistematização dos Cuidados à Saúde da Criança; Vivenciando a SAE na Saúde da Criança; Pactuação do Protocolo de Saúde da Criança. Os encontros proporcionaram reflexão, discussão, compartilhamento e compreensão entre a equipe de enfermagem durante esse processo.

A escolha por oficinas representa uma alternativa na busca por melhorias, constituindo um espaço de tempo complexo onde os participantes são atores e sujeitos que criam formas de interação que podem transcender a aplicação crítica de teorias e práticas. Ao todo, foram realizadas quatro oficinas, sendo duas remotas e duas presenciais, além de encontros formais e não formais.

A primeira oficina caracterizou a Fase de Reconhecimento, que precedeu o início do grupo. O objetivo foi apresentar o projeto de pesquisa aos profissionais participantes. A apresentação do grupo ocorreu de forma remota.

A segunda oficina representou a Fase de Revelação. O objetivo da segunda oficina foi desenvolver caminhos e consensos com os participantes para sistematizar os cuidados à saúde da criança conforme o protocolo definido.

Na terceira oficina, abordou-se a Fase do Repartir. Esta oficina aconteceu presencialmente na UBS, durou cerca de 30 minutos e teve como objetivo compartilhar a experiência do protocolo com a prática diária. Os participantes foram divididos em grupos para não interferir na rotina.

Na quarta e última oficina, definiu-se a Fase do Repensar. Esta oficina ocorreu na UBS e durou cerca de 30 minutos. Permitindo uma reflexão sobre o conteúdo do processo por meio de discursos e consensos com os profissionais de enfermagem, após utilizá-lo durante a CE na unidade.

De um total de nove profissionais, uma equipe estava sem enfermeira por estar de licença. A primeira autora é enfermeira na UBS. Dessa forma, participaram sete profissionais de enfermagem, sendo duas enfermeiras e cinco técnicos de enfermagem. Os critérios de inclusão foram: profissionais de enfermagem que prestavam assistência à criança na UBS cenário do presente estudo. E, como critério de exclusão: profissionais de enfermagem em licença por diferentes motivos durante o período da pesquisa. Os encontros formais foram definidos como aqueles realizados por meio do aplicativo *Microsoft Teams* e no auditório da UBS. Já os encontros não formais aconteceram durante os intervalos das CEs, na UBS, e por meio da utilização da ferramenta WhatsApp. O processo de implantação acontecia simultaneamente ao processo de adaptação que estava sendo discutido no grupo pelos profissionais participantes.

### A Quinta Fase: Avaliação

Os resultados foram analisados e discutidos simultaneamente às oficinas de implantação, sendo registrados em um diário de campo, por meio de observação participante, e nas falas dos participantes, através do compartilhamento de experiências, vivências e sugestões no processo de implantação. Avaliaram-se as percepções dos participantes em relação ao protocolo e ao processo de adaptação, utilizando o *Google Forms*. Ao analisar os resultados, percebeu-se uma opinião favorável à implantação do Protocolo de Enfermagem. Por fim, a versão final foi encaminhada para avaliação pelo Departamento de Atenção Básica da Secretaria Municipal de Saúde, sendo considerada aprovada para implantação nas UBS do município.

### Análise

A proposta metodológica de análise seguiu o referencial proposto por Creswell^(13)^. Posteriormente, os dados discutidos nas oficinas foram transcritos na íntegra conforme o desenvolvimento de cada oficina, sendo identificados e analisados conforme o tema e os objetivos propostos, utilizando o software Word. Cada participante foi identificado por letras e números, para assegurar o anonimato.

A síntese teve como objetivo comparar os conceitos extraídos das falas, utilizando cores distintas para cada participante, de acordo com os conceitos identificados. Os dados foram comparados, contextualizados e ordenados segundo os relatos dos participantes sobre ideias, concepções, eventos e fatos.

Considerando os valores encontrados nas informações coletadas durante a síntese, surgiram quatro categorias. Dessa forma, para cada categoria, derivada da análise aprofundada, que representa o sentido extraído, a discussão foi sustentada com base na literatura científica.

## RESULTADOS

Para a adaptação do protocolo, foi necessário explorar o tema com o grupo para que este pudesse oferecer suporte teórico e prático entre os profissionais de saúde. Dos participantes, a prevalência foi o sexo feminino, com seis mulheres e um homem. O tempo de atuação no cargo e na UBS variou: um dos profissionais tinha mais de 30 anos, quatro tinham mais de 20 anos, e dois tinham entre 5 e 15 anos de experiência na área de saúde, com uma média de tempo de trabalho na ESF de 24,2 anos. O tempo de experiência variou de 24 meses a 35 anos.

Após a comparação das informações identificadas, foram estabelecidas quatro categorias: Experiências e vivências com o protocolo no dia a dia; Fragilidades e limitações no processo de implantação; Facilidades no processo de implantação; e Sugestões para complementar o protocolo para a realidade de RO.

Sob a ótica dos participantes da pesquisa, a categoria intitulada “Experiências e vivências com o protocolo no dia a dia” implica na melhoria da qualidade dos serviços de saúde ofertados para as crianças na APS, possibilitando uma melhor integração da equipe, resultando no acolhimento da mãe/usuária e de sua criança, na realização da CE, na humanização da assistência, no aumento da demanda reprimida e, por fim, na integração teórica com a prática.

A categoria “Fragilidades e limitações no processo de implantação do protocolo” refere-se ao impacto cotidiano das demandas durante as CEs, assim como às limitações do acesso no âmbito do SUS, enfatizando o contexto, as estratégias estabelecidas, alguns dos seus determinantes e condicionantes, o dimensionamento inadequado da equipe de enfermagem, além do pouco tempo para o estudo e a preocupação em expandir o protocolo para todas as unidades básicas de saúde.

Já a categoria “Facilidades no processo de implantação” descreve o protocolo como uma ferramenta facilitadora na implementação da sistematização da assistência de enfermagem (SAE), promovendo a melhoria dos processos de trabalho em busca da excelência assistencial. Além disso, a utilização de recursos audiovisuais como suporte ao processo e a adaptação do protocolo já validado pelo COREN/PR efetivamente facilitaram a implantação.

Por fim, a categoria “Sugestões para complementar o Protocolo para a realidade do município” discorre sobre o compartilhamento do conhecimento e a análise da percepção dos profissionais de enfermagem na melhoria do produto final. Assim sendo, o processo de adaptação do Protocolo de Enfermagem na APS: Módulo 4 - COREN/PR, foi ajustado para a realidade da unidade de saúde em Ji-Paraná.

### Processo de adaptação do protocolo

Com o processo de adaptação, obteve-se um novo produto. Este foi reestruturado para a realidade local com a contribuição dos profissionais por meio dos encontros. O protocolo original continha oito capítulos, dos quais todos sofreram adaptações, sendo ainda incluídos subcapítulos e 11 anexos, conforme o Quadro 1.

**Quadro 1:** Comparativo entre protocolo original e protocolo adaptado, Ji-Paraná, Rondônia, Brasil, 2023

Protocolo Original	Protocolo Adaptado
1. Consulta do Enfermeiro para acompanhamento do crescimento e desenvolvimento da criança sadia. 2. Consulta do Enfermeiro para acompanhamento do crescimento e desenvolvimento da criança com demandas diferenciadas de cuidado 3. Avaliação da criança com problemas respiratórios 3.1 Dor de ouvido 4. Avaliação da criança com problemas de nutrição ou de alimentação 5. Avaliação da criança com diarreia e/ou desidratação 6. Avaliação da criança com anemia. 7. Avaliação da criança com problemas oculares 8. Avaliação da criança com problemas dermatológicos	1. Consulta do Enfermeiro para acompanhamento do crescimento e desenvolvimento da criança sadia. 2. Consulta do Enfermeiro para acompanhamento do crescimento e desenvolvimento da criança com demandas diferenciadas de cuidado 3. Avaliação da criança com problemas respiratórios 3.1 Dor de ouvido 4. Avaliação da criança com problemas de nutrição ou de alimentação 5. Avaliação da criança com diarreia e/ou desidratação 5.1 Procedimentos a serem adotados em caso de disenteria e/ou outras patologias associadas à diarreia 5.2 Vigilância epidemiológica das Doenças Diarreicas Agudas (DDA) 6. Avaliação da criança com anemia. 6.1 Diagnóstico laboratorial 6.2 Tratamento 7. Avaliação da criança com problemas oculares 8. Avaliação da criança com problemas dermatológicos

O Quadro 2 descreve de forma sucinta as adaptações e justificativas de cada item de acordo com cada capítulo.

**Quadro 2:** Síntese do protocolo adaptado, Ji-Paraná, Rondônia, Brasil. 2023

Capítulo 1. Consulta do Enfermeiro para acompanhamento do crescimento e desenvolvimento da criança sadia	
**Incluído na adaptação**	**Justificativa**
As atribuições específicas dos profissionais de enfermagem e agentes comunitários de saúde em relação à criança.	Deixar claro as atribuições de cada profissional
Inseridos os quadros: Representação dos reflexos da criança; representação dos testes de Barlow e Ortolani; Consultas da Criança - Baixo Risco; Atualização da estratificação de risco; ampliação da solicitação de exames.	Enriquecer e facilitar o acesso às informações, além de deixar claro os exames que os profissionais podem solicitar.
Acrescentado no Quadro 9: Eventos Adversos Pós-Vacinação; Obstrução nasolacrimal congênita; hérnia umbilical; hérnia inguinal; criptorquidia; hidrocele; fimose; Coto umbilical; granuloma umbilical; refluxo; A conduta do granuloma umbilical na ausência de bastão de nitrato de prata.	Facilitar a conduta dos profissionais
**Capítulo 2. Consulta do Enfermeiro para acompanhamento do crescimento e desenvolvimento da criança com demandas diferenciadas de cuidado**	
Inserido o Quadro Classificação do Recém-Nascido. Fluxograma Consulta de Enfermagem à criança com desidratação	Facilitar o acesso às informações
**Capítulo 3. Avaliação da criança com problemas respiratórios**	
Inserido os Quadros: Avaliação da dificuldade para respirar; Condutas para tosse ou dificuldade para respirar.	Facilitar o acesso às informações
**Capítulo 3.1 Dor de ouvido**	
Inserido os Quadros: Avaliação e classificação das crianças com dor de ouvido; Achados à otoscopia	Facilitar o acesso às informações
**Capítulo 4. Avaliação da criança com problemas de nutrição ou de alimentação**	
Inserido os Quadros: Avaliação da desnutrição conforme AIDPI Brasil^(14)^ Condutas diante do crescimento da criança; Profilaxia - Administração de Vitamina A em Crianças; Ganho Antropométrico - 1º ao 2º ano.	Facilitar o acesso às informações
**Capítulo 5.** Avaliação da criança com diarreia e/ou desidratação	
Inserido os Quadros: Características diarreicas menores de 2 anos a partir do texto descrito pelos autores. Condutas durante o tratamento em crianças.	Facilitar o acesso às informações
**Capítulo** 6. Avaliação da criança com anemia.	
O texto logo no início do capítulo foi resumido. O fluxograma 7 foi adaptado para o local.	Facilitar o acesso às informações
**Capítulo** 7. Avaliação da criança com problemas oculares	
Inserido o quadro - Problemas Oculares	Facilitar o acesso às informações
**Capítulo** 8. Avaliação da criança com problemas dermatológicos	
Monilíase oral com imagens; Intertrigo eritematoso; Micose; *Tínea Corporis;* Picadas de Inseto Estrófulo; Varicela; Síndrome Mão - Pé - Boca; Impetigo não Bolhoso; Miíase furunculóide; Larva Migrans	Facilitar o acesso às informações
**Anexos do Protocolo**	
Curva de Crescimento por idade do nascimento aos 5 anos; Marcos do desenvolvimento nos primeiros 5 anos; Intercorrências e condutas para a puericultura em situações especiais; Calendário de vacinação de rotina do Ministério da Saúde; Icterícia Neonatal; Promovendo a Saúde Bucal.	Facilitar o acesso às informações

*AIDPI - Atenção Integrada às Doenças Prevalentes na Infância.*

## DISCUSSÃO

Os protocolos visam contribuir para cuidados adequados e eficazes, garantindo efetividade para a condição clínica, proporcionando mais vantagens do que danos. Além disso, visam auxiliar na tomada de decisão, constituindo uma situação singular de cuidado com detalhes e especificações operacionais^(15)^. Nesse sentido, ao identificarmos, na fase de diagnóstico, a precariedade na tomada de decisão do cuidado padronizado, chegamos ao consenso de que os profissionais devem seguir protocolos baseados em evidências ao cuidar de crianças.

Os protocolos estão se tornando instrumentos amplamente utilizados no fomento da enfermagem baseada em evidências, permitindo ao enfermeiro implementar o conhecimento científico na assistência, qualificando-a e tornando-a segura^(16)^. Existem vários modelos de protocolos, com enfoque na tecnologia leve-dura, organizados de acordo com seu conteúdo prático. A maior diferença entre eles é a sua aplicabilidade^(15)^. Na fase de investigação, constatamos que apenas onze protocolos foram validados e possuíam acesso à assistência oportunizada. Após a avaliação dos elementos relacionados à elegibilidade, os documentos disponíveis foram verificados e a aprovação para a implantação foi concedida. Na terceira fase, após a coleta de dados e análise profunda, foi definido o protocolo a ser adaptado.

A fase de implantação envolveu a realização de oficinas temáticas usando o método Quatro Erres (RS). Como resultado, viabilizou-se a adaptação do protocolo. A literatura indica que a utilização de protocolos beneficia o desempenho dos enfermeiros, conduzindo suas condutas terapêuticas. Essa observação se fundamenta na habilidade dos protocolos de resumir o processo terapêutico, associado à diminuição do tempo gasto na execução do registro da conduta profissional, complementando a sistematização das informações disponibilizadas para um cuidado de enfermagem contínuo^(17)^.

Dessa maneira, os protocolos assistenciais e de cuidados, procedimentos operacionais padrão, entre outros, são instrumentos para orientar os enfermeiros no cuidado aos pacientes, devendo ser aplicados na realidade dos diversos cenários de atenção à saúde para proporcionar uma assistência de qualidade^(18)^.

Nesse sentido, durante as oficinas, a equipe de enfermagem foi sensibilizada sobre a importância do tema, recebeu explicações sobre como o protocolo seria aplicado e, após começar a utilizá-lo, durante as consultas, houve discussões periódicas com a pesquisadora sobre dúvidas, pontos negativos, pontos positivos e sugestões.

Em uma revisão sistemática, observou-se que poucos estudos científicos identificam e avaliam as habilidades dos enfermeiros em cuidados de saúde da criança. A pesquisa incluiu 18 estudos de seis países e descreveu as habilidades clínicas dos enfermeiros na infância. No entanto, os resultados não mostram uma base sólida para avaliar o alcance de suas práticas. Educação e aconselhamento em saúde, avaliação da saúde e desenvolvimento de crianças e adolescentes, imunizações e exames de saúde infantil foram as competências de enfermagem mais comuns identificadas, descritas e realizadas de forma independente^(19)^.

No que concerne à interação dos profissionais, durante o processo de implantação do protocolo, os participantes da pesquisa, entusiasmados, trouxeram sugestões e colocaram em prática o proposto no protocolo nas CE. Assim, ao identificar uma situação real, durante as CE em saúde da criança, não descrita no protocolo, o profissional a anotava e, em seguida, todas as dúvidas eram discutidas em equipe, visando à adequação do protocolo como um processo de tomada de decisão conjunta. Isso resultou em um relacionamento entre os membros da equipe e os usuários, promovendo uma comunicação dialógica, empática e ética, capaz de estimular a autonomia, a cooperação e a corresponsabilização dos moradores, contribuindo na prática, conforme evidenciado em um estudo realizado em Recife-PE^(20)^.

Durante esse período, observou-se, por meio de contatos informais com toda a equipe de saúde do serviço, o resultado das consultas norteadas pelo protocolo. A aplicação do protocolo nas CE aumentou a demanda por serviços de enfermagem na unidade. Observa-se, portanto, que o planejamento participativo realmente contribuiu para a mudança na UBS em que foi implementado. Além disso, tem potencial de promover mudanças institucionais em todo o setor saúde do município no que diz respeito à saúde da criança.

Esse fato corrobora com o encontrado na literatura, visto que a busca por qualidade no cuidado nas ações dos enfermeiros e a construção coletiva dialogada impulsionam o desenvolvimento de protocolos locais. Nessa perspectiva, reconhecer os protocolos como ferramentas de apoio à decisão, que conferem suporte técnico e ético, contribui para a construção de conhecimento e para o desenvolvimento de boas práticas em enfermagem^(21)^.

Sob esse ponto de vista, a fase de avaliação, que consiste em um processo de construção coletiva e dialogada, bem como a adequação dos protocolos à realidade local, constituíram um importante exercício de educação permanente e aprofundamento dos conhecimentos técnico-científicos dos enfermeiros, com a participação ativa dos diversos atores no processo de aprendizagem^(21)^.

Assim, fica explícito neste estudo o quanto os profissionais compreendem que o estabelecimento de protocolos de cuidados auxilia na organização e estruturação dos processos de trabalho, promovendo a melhoria da qualidade da assistência.

Nesse contexto, é preciso refletir e repensar sobre a gama de produtos, como a construção de protocolos, importantes para o cuidado de enfermagem. Além disso, é válido destacar o rico processo de implantação, que mescla conhecimentos teóricos, vivências e experiências práticas, como uma tendência a ser desenvolvida no âmbito do trabalho, pois esse processo requer competência dos enfermeiros que atuam na APS para orientar sua prática.

### Limitações do estudo

Como limitação do estudo, podem ser citados alguns pontos importantes. Destacam-se: a pandemia de SARS-CoV-2, com todas as barreiras e medidas de distanciamento social para evitar a propagação da COVID-19. Durante a fase mais crítica da pandemia, a unidade continuou funcionando, e as oficinas, inicialmente programadas para acontecer de forma presencial, foram reorganizadas para o formato remoto. Outro fator limitante foi a falta de horários disponíveis no trabalho para que os profissionais de enfermagem se reunissem, além da sobrecarga de trabalho.

### Contribuições para a Área

A adaptação do protocolo à realidade local trouxe benefícios para o cuidado em saúde e enfermagem, como maior segurança para o enfermeiro e aumento no número de consultas de enfermagem para a saúde da criança. O protocolo de enfermagem promove conhecimento, habilidade e atitude profissional, possibilitando a adoção de práticas baseadas em evidências e proporcionando uma assistência segura e qualificada às crianças na APS da Região Amazônica, com impactos positivos em seu crescimento e desenvolvimento.

## CONSIDERAÇÕES FINAIS

A adaptação e implantação do protocolo de cuidados de enfermagem para a saúde das crianças na APS foram bem-sucedidas, trazendo benefícios significativos. Esse processo permitiu que os enfermeiros, em conjunto com a equipe, compartilhassem conhecimento científico, favorecendo uma assistência segura, qualificada e baseada em evidências.

Evidenciamos melhorias em cada etapa da aplicação do protocolo no cuidado de enfermagem, resultando em cuidados de saúde melhores para as crianças, integração da equipe e melhor atendimento para mãe e filho. A pesquisa metodológica ajustou o protocolo à realidade local e facilitou a implantação, coletando informações durante oficinas temáticas. Inicialmente, foi implantado em uma unidade de saúde, mas demonstrou alto potencial para ser institucionalizado a nível municipal, devido à sua aplicabilidade prática.

As atividades de acompanhamento da criança, o aprendizado e a experiência adquirida após as oficinas foram confirmados pelo apoio positivo dos participantes. Demonstramos que o processo de adaptação e implantação é uma abordagem eficaz para melhorar o cuidado, fortalecendo a enfermagem como profissão com bases sólidas em evidências científicas e clínicas. Isso permite a identificação precoce de problemas e a orientação adequada, resultando em melhores desfechos de saúde para a criança. Espera-se que este estudo contribua para a criação de novos dados e evidências científicas no campo.
